# Depth Filters Containing Diatomite Achieve More Efficient Particle Retention than Filters Solely Containing Cellulose Fibers

**DOI:** 10.3389/fpls.2015.01134

**Published:** 2015-12-21

**Authors:** Johannes F. Buyel, Hannah M. Gruchow, Rainer Fischer

**Affiliations:** ^1^Integrated Production Platforms, Fraunhofer-Institute for Molecular Biology and Applied Ecology IMEAachen, Germany; ^2^Institute for Molecular Biotechnology, RWTH Aachen UniversityAachen, Germany

**Keywords:** bioprocess costs, clarification and filtration, design of experiments, model building, plant-derived biopharmaceuticals, pre-coat filtration

## Abstract

The clarification of biological feed stocks during the production of biopharmaceutical proteins is challenging when large quantities of particles must be removed, e.g., when processing crude plant extracts. Single-use depth filters are often preferred for clarification because they are simple to integrate and have a good safety profile. However, the combination of filter layers must be optimized in terms of nominal retention ratings to account for the unique particle size distribution in each feed stock. We have recently shown that predictive models can facilitate filter screening and the selection of appropriate filter layers. Here we expand our previous study by testing several filters with different retention ratings. The filters typically contain diatomite to facilitate the removal of fine particles. However, diatomite can interfere with the recovery of large biopharmaceutical molecules such as virus-like particles and aggregated proteins. Therefore, we also tested filtration devices composed solely of cellulose fibers and cohesive resin. The capacities of both filter types varied from 10 to 50 L m^−2^ when challenged with tobacco leaf extracts, but the filtrate turbidity was ~500-fold lower (~3.5 NTU) when diatomite filters were used. We also tested pre–coat filtration with dispersed diatomite, which achieved capacities of up to 120 L m^−2^ with turbidities of ~100 NTU using bulk plant extracts, and in contrast to the other depth filters did not require an upstream bag filter. Single pre-coat filtration devices can thus replace combinations of bag and depth filters to simplify the processing of plant extracts, potentially saving on time, labor and consumables. The protein concentrations of TSP, DsRed and antibody 2G12 were not affected by pre-coat filtration, indicating its general applicability during the manufacture of plant-derived biopharmaceutical proteins.

## Introduction

The successful launch of Elelyso in 2012 by Protalix Biotherapeutics (Carmiel, Israel) (Tekoah et al., 2015) showed that plants and plant cells are competitive expression systems for biopharmaceutical proteins (Buyel, 2015; Mor, 2015). The production of biopharmaceutical proteins in plants offers distinct benefits such as a low pathogen burden (Commandeur and Twyman, 2005) but also challenges such as low expression levels (2–50 mg kg^−1^ biomass) (Twyman et al., 2005). Cost-effective production can be difficult in plants compared to established platforms based on mammalian cells, and this makes it harder to achieve commercialization. The costs associated with upstream production (USP) in plants are often low, especially for open-field cultivation (Stoger et al., 2002), but up to 80% of the total production costs are attributed to downstream processing (DSP) (Wilken and Nikolov, 2012; Buyel et al., 2015).

Traditionally, DSP is divided into primary recovery and purification (Menkhaus et al., 2004). Like all other platforms, the purification of plant-derived proteins is based on the intrinsic properties of the target and can be achieved with standard operations such as chromatography (Buyel and Fischer, 2014a). In contrast, primary recovery requires specific clarification steps such as depth filtration or flocculation to address issues that are specific to plants, such as the high particle burden in the feed stream (Buyel and Fischer, 2014b). The particle burden can be reduced if the product is secreted or specialized extraction methods are used, e.g., guttation (Komarnytsky et al., 2000), rhizosecretion (Drake et al., 2009) and infiltration-centrifugation (Turpen, 1999), but these techniques are limited to secreted proteins. Typically, the product accumulates in the plant tissue and must be released from intracellular compartments by homogenization (Hassan et al., 2014; Buyel and Fischer, 2014c,d). The use of blade-based homogenizers releases large amounts of dispersed particles producing extracts with turbidities exceeding 5000 nephelometric turbidity units (NTU) (Buyel and Fischer, 2014e).

Centrifuges can be used for clarification but single-use filters are preferred because these are less expensive, more scalable and do not require cleaning validation (Roush and Lu, 2008; Pegel et al., 2011; O'Brien et al., 2012). Filters can also remove host cell proteins (HCPs) and pigments (Yigzaw et al., 2006; Naik et al., 2012). Single-use depth filters have been identified as the major consumables cost-driver during DSP (Buyel et al., 2014) so additives such as flocculants and filter aids have been tested to improve filter capacity and thus reduce costs (Buyel and Fischer, 2014f). Although these additives are effective, they can also encourage the precipitation of the target protein (Holler et al., 2007), they may be incompatible with subsequent downstream operations (Buyel and Fischer, 2014b), and they may increase safety risks (Buyel and Fischer, 2014e). Therefore, single-use filters that increase filter capacity but use only harmless and easily-removed additives are preferred, and once identified they can reduce DSP costs and thus to improve the economic competitiveness of molecular pharming.

**Figure 1:**
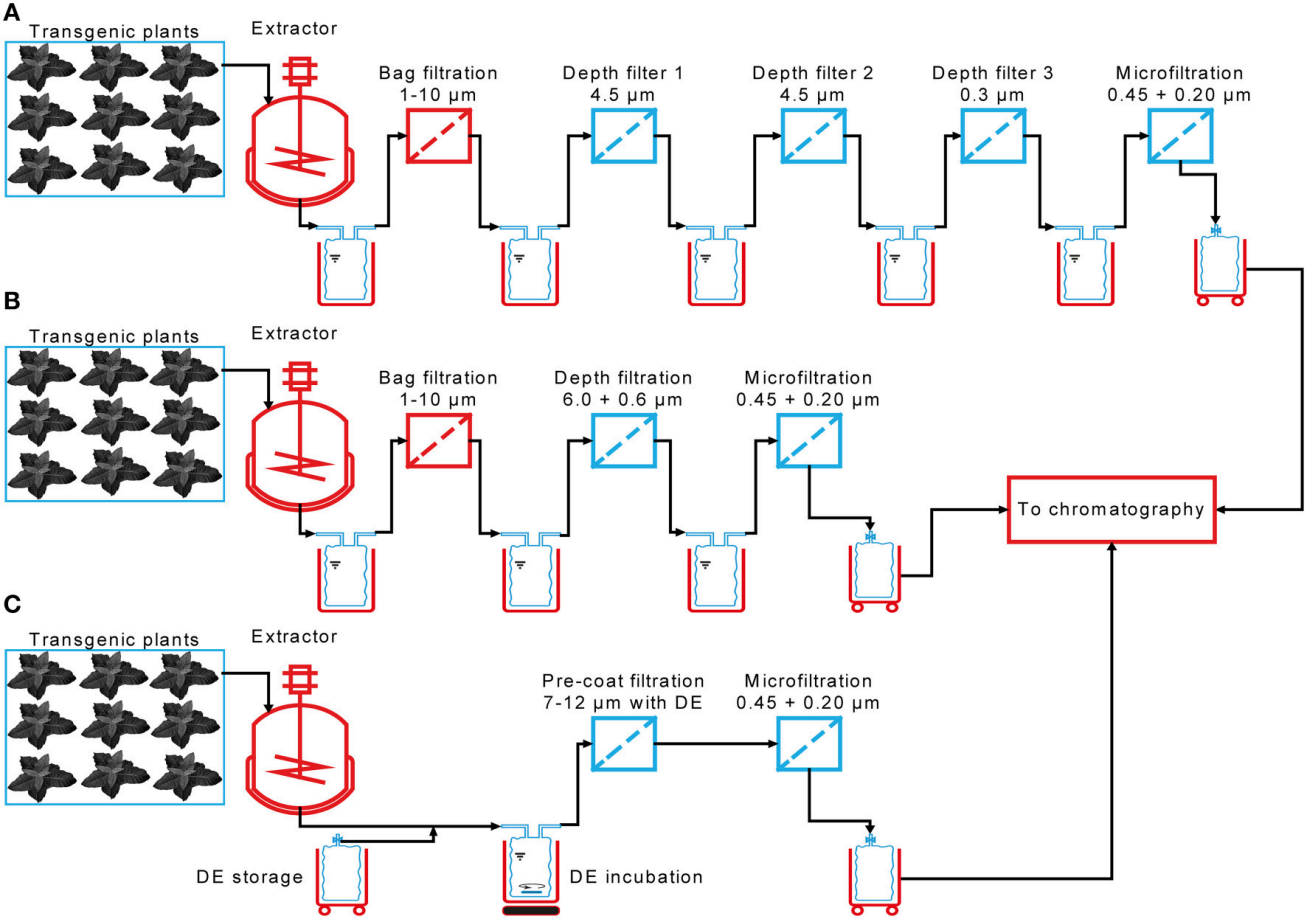
**Incremental reduction in the complexity of a process for plant extract clarification**. **(A)** The clarification process implemented for the manufacture of the plant-derived antibody 2G12 under GMP conditions in 2009 consisted of a total of five filtration steps, including three depth filters. **(B)** The filter train complexity was reduced as part of a new production campaign in 2013/2014, now consisting of three filtration steps and two multi-use components. **(C)** In the current process, a pre-coat filtration step replaces the bag and depth filters, reducing both the number of filtration steps and the number of multi-use components.

Here we compare the performance of 24 different depth filters containing diatomaceous earth (DE) to a reference filter train used during the manufacture of a plant-derived monoclonal antibody in an 800-L scale process (Figure 1) compliant with good manufacturing practice (GMP). We also tested seven filter combinations lacking DE, which can be beneficial if target proteins bind to this charged filter aid. We investigated the scalability of small filtration devices, and finally used a design of experiments (DoE) approach to characterize a DE pre-coat filtration technology that can potentially simplify tandem filtration systems consisting of a bag filter and a depth filter in series, reducing this to a single clarification unit (Figure 1). The performance of all filters was tested using plant extracts containing two model target proteins: the fluorescent protein DsRed and monoclonal antibody 2G12.

## Materials and methods

### Biological materials

Seeds of transgenic tobacco (*Nicotiana tabacum*) line pGFD (Buyel and Fischer, 2014c) were germinated in soil and cultivated in a greenhouse at 25/22°C day/night temperature with 70% relative humidity. The plants were irrigated with 0.1% (w/v) Ferty 2 Mega (Kammlott GmbH, Germany) for 15 min h^−1^ during a 16-h photoperiod (180 μmol s^−1^ m^−2^; λ = 400−700 nm) and were grown for 50–53 days prior to harvest.

### Extraction and filtration

Three volumes (1500 mL) of extraction buffer (50 mM sodium phosphate, 500 mM sodium chloride, 10 mM sodium disulfite, pH 8.0) were added to 500 g of plant material and homogenized in a PT 6100 (Kinematica, Switzerland) customized with a blade tool and a 3-L vessel. Prior to depth filtration, the extract was pre-clarified using a BP-410 bag filter (Fuhr, Klein-Winternheim, Germany) with a nominal retention rating of 1 μm. The capacity and efficiency of different depth filters (Table 1) with an area of 22-23 cm^2^ were tested at a constant volumetric loading flow rate of 12 mL min^−1^ using a PDF4 filter (Pall, Dreieich, Germany) as a reference (Buyel and Fischer, 2014c). Turbidities were determined at each process step using a 2100P turbidimeter (Hach, Loveland, CO, USA) as 1:40 (homogenate), 1:10 (bag filtrate, depth filters after particle breakthrough) or undiluted samples (regular depth filtrates, pre-coat filtrates). Conductivity and pH were also monitored at each process step. We also tested filters PDH4 and PDF4 in a Supracap format with authentic filter layer geometry under the same conditions. Depth filters were also compared with single-use Sartolab DY (DY) pre-coat filters with a filter area of 22 cm^2^ (Sartorius, Göttingen, Germany) which were operated at 1500 Pa vacuum using a N816.3KN.18 membrane pump (KNF, Freiburg, Germany) and 150 mL of extract or bag filtrate, with or without 2.0 g L^−1^ of the flocculant Polymin P (BASF, Ludwigshafen, Germany). In these initial tests 140 g L^−1^ DE Celpure C300 (Sartorius) was added to the feed before filtration. The results were confirmed using an I-optimal DoE consisting of 14 runs using a scalable custom filter housing (Sartoclear Dynamics (SD), Sartorius) equipped with a Purex filter layer (Sartorius, nominal retention rating of 7-12 μm, 12.5 cm^2^ filter area) and operated with a feed flow rate of 6.3 mL min^−1^. In the DoE setup, DE concentrations of 25-60 g L^−1^, pre-filtration incubation times of 10–90 min and either one or two additions of DE were investigated using bulk plant extract at all times. Particle size distributions were determined with a Zetasizer NanoZS (Malvern, Malvern, UK) using undiluted samples.

**Table 1:** **Depth filters tested for the clarification of bag-filtered plant extract in ascending order of retention rating**.

**Name of**				**Number of**			**Average retention rating [**μ**m] of**		
**First layer**	**Second layer**	**Manufacturer**	**Filter code**	**Filters**	**Layers**	**Filter area [cm^2^]**	**First filter**	**Second filter**	**Retention number RN [-]**
*Bio20*	*Bio10*	*Pall*	*P3*	*1*	*2*	*22*	*0.7*	*0.3*	*1.17*
AF101H	CHST150P	Filtrox	F1	1	2	22	1.05	0.12	4.38
AF101H	AFSTI40	Filtrox	F2	1	2	22	1.05	0.30	1.75
AF101H	CHST110P	Filtrox	F3	1	2	22	1.05	0.65	0.81
*CE45*	*CE50*	*Merck/Milllipore*	*M3*	*2*	*2*	*23*	*1.45*	*0.85*	*0.85*
*CE35*	*CE50*	*Merck/Milllipore*	*M4*	*2*	*2*	*23*	*1.75*	*0.85*	*1.03*
*CE40*	*CE50*	*Merck/Milllipore*	*M5*	*2*	*2*	*23*	*2.00*	*0.85*	*1.18*
CH71HP	CHST150P	Filtrox	F4	1	2	22	2.25	0.12	9.38
CH71HP	AFST140	Filtrox	F5	1	2	22	2.25	0.30	3.75
CH71HP	CHST110P	Filtrox	F6	1	2	22	2.25	0.65	1.73
*CE30*	*CE50*	*Merck/Milllipore*	*M6*	*2*	*2*	*23*	*3.75*	*0.85*	*2.21*
PB2	PC2	Sartorius	S1	2	4	25	4.50	0.30	1.25
D0HC	B1HC	Merck/Milllipore	M1	2	4	23	4.80	0.40	1.00
D0HC	C0HC	Merck/Milllipore	M2	2	4	23	4.80	1.10	0.36
3M 39662	3M 41082	3M	3M1	2	4	25	6.00	0.45	1.11
**K200**	**KS50**	**Pall**	**PDF4**	**1**	**2**	**22**	**6.00**	**0.60**	**5.00**
**PDF4 SupraCap**		**Pall**	**P1S**	**1**	**2**	**26**	**6.00**	**0.60**	**5.00**
3M 39662	3M 41102	3M	3M2	2	4	25	6.00	0.63	0.80
*CE25*	*CE50*	*Merck/Milllipore*	*M7*	*2*	*2*	*23*	*6.00*	*0.85*	*3.53*
CH50P	CHST150P	Filtrox	F7	1	2	22	6.30	0.12	26.25
CH50P	AFST140	Filtrox	F8	1	2	22	6.30	0.30	10.50
CH50P	CHST110P	Filtrox	F9	1	2	22	6.30	0.65	4.85
CH41HP	CHST150P	Filtrox	F10	1	2	22	6.50	0.12	27.08
CH41HP	AFST140	Filtrox	F11	1	2	22	6.50	0.30	10.83
CH41HP	CHST110P	Filtrox	F12	1	2	22	6.50	0.65	5.00
*CE20*	*CE50*	*Merck/Milllipore*	*M8*	*2*	*2*	*23*	*8.00*	*0.85*	*4.71*
CH31HP	CHST150 P	Filtrox	F13	1	2	22	8.50	0.12	35.42
CH31HP	AFST140	Filtrox	F14	1	2	22	8.50	0.30	14.17
CH31HP	CHST110P	Filtrox	F15	1	2	22	8.50	0.65	6.54
AF21H	CHST150P	Filtrox	F16	1	2	22	10.50	0.12	43.75
AF21H	AFST140	Filtrox	F17	1	2	22	10.50	0.30	17.50
AF21H	CHST110P	Filtrox	F18	1	2	22	10.50	0.65	8.08
K700	KS50	Pall	P2	1	2	22	11.00	0.60	9.17
**PDH4 SupraCap**		**Pall**	**P2S**	**1**	**2**	**26**	**11.00**	**0.60**	**9.17**

*Filters without diatomaceous earth are shown in italics, and filters with scalable geometry are shown in bold. The filter used in our standard process is bold underlined. Filters achieving a similar capacity are underlined. The filter code is used in all subsequent figures for brevity*.

### Protein quantitation

Samples from extracts and filtrates were centrifuged twice (16,000 × g, 20 min, 4°C) and the quantity of total soluble protein (TSP) in the supernatants was determined using the Bradford method (Simonian and Smith, 2006) adapted to a microtiter plate format (Buyel and Fischer, 2014f) with a triplicate standard curve of eight dilutions of bovine serum albumin in the range 0–2000 μg mL^−1^. The absorbance at 595 nm was measured for technical triplicates of each sample using a Synergy HT plate reader (BioTek Instruments, Vermont, USA). The same reader fitted with a 530/25 nm (excitation) and 590/35 nm (emission) filter set was used to quantify the concentrations of DsRed in the supernatants. A standard curve was generated with six dilutions of purified DsRed in the range of 0–225 μg mL^−1^. Concentrations of antibody 2G12 were determined by surface plasmon resonance (SPR) spectroscopy using an SPR2 instrument (Sierra Sensors, Hamburg, Germany). For each sample, the concentration of 2G12 was measured by binding to protein A, which was immobilized on the surface of a high capacity amine chip (Sierra Sensors) by EDC/NHS coupling, and comparison to a 1.0 μg mL^−1^ reference solution of 2G12 used for one-point calibration (Buyel and Fischer, 2014c). HBS-EP+ (10 mM HEPES, pH 7.4, 150 mM NaCl, 3 mM EDTA, 0.05% v/v Tween-20) was used as the running buffer.

## Results

### Clarification of plant extracts using conventional depth filters

**Figure 2:**
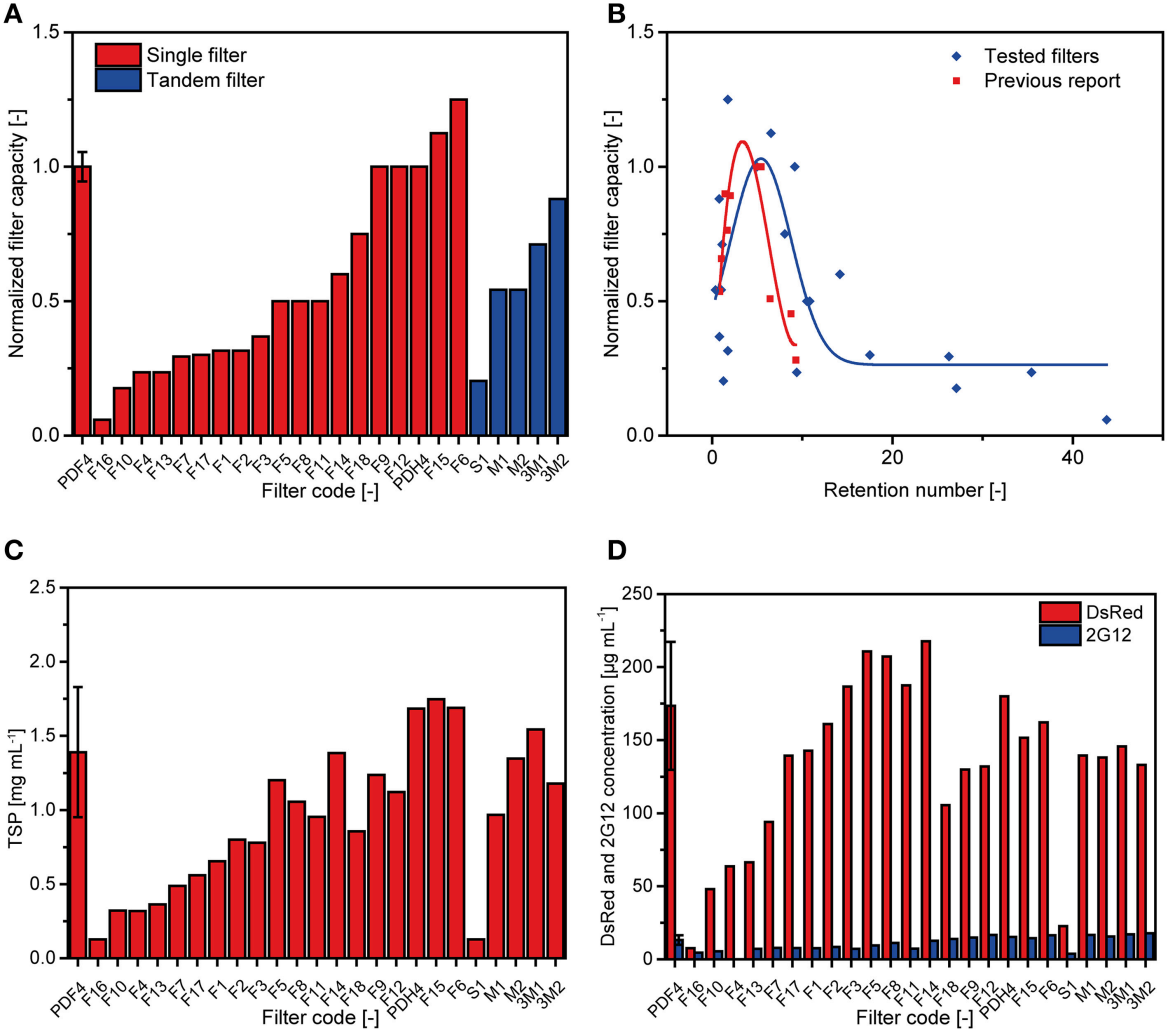
**Performance of conventional depth filters in terms of capacity and protein binding**. **(A)** The capacity of 24 conventional depth filter setups (single filter or tandem filter) was tested in five batches and normalized to two runs of the reference filter P1 (PDF4) included in each batch (Buyel and Fischer, 2014c). **(B)** Normalized capacity over *RN* plot showing the results for the 25 filters (including the reference) tested here (blue) compared to a previous report (red) (Buyel and Fischer, 2014c). The maximum of a Gaussian function fitted to the data (blue line) indicates the theoretical optimum for *RN* and capacity. A cubic fit to the previously reported data is shown for comparison (red line). **(C)** TSP concentrations in the filtrates of the different filter setups as determined by the Bradford method. **(D)** Concentrations of DsRed and 2G12 were determined in the different filtrates by fluorescence spectroscopy and SPR spectroscopy, respectively. Error bars indicate the standard deviation of all reference runs (*n* = 10).

We tested 24 conventional depth filter setups containing DE, 19 of which consisted of a single filter (two filter layers) and five as tandem filters (four filter layers). The test was conducted in five runs and capacities were normalized to a PDF4 reference filter (Figure 1B) which achieved a capacity of 35 ± 6 L m^−2^ (*n* = 10; two per run; Figure 2A). Filters F6 and F15 outperformed PDF4 in terms of capacity by 25 and 13%, respectively. However, the turbidities of the F6 and F15 filtrates were 14 and 23 NTU, respectively, compared to 4 ± 2 NTU (*n* = 10) for PDF4. None of the tandem filters we tested achieved capacities greater than that of PDF4.

A dimensionless retention number (*RN*) has previously been used to pre-select filter layer combinations that are likely to achieve high capacities (Equation 1) (Buyel and Fischer, 2014c).

(1) $$\begin{array}{l} RN=\frac{\frac{r_{1}}{r_{2}}+\frac{r_{2}}{r_{3}}+\cdots +\frac{r_{i}}{r_{j}}+\cdots +\frac{r_{n-1}}{r_{n}}}{\left(n-1\right)\times n} \end{array}$$

where *n* is the total number of layers, *r*_*i*_ is the nominal retention rating of the more porous layer in each pair of consecutive layers, and *r*_*j*_ is the nominal retention rating of the finer layer in the pair. We calculated the *RN* for the 24 filters tested here and found that the average *RN* of the five new filters with the highest normalized capacity, i.e., ≥1.0, was 5.9 ± 2.7 (*n* = 5), whereas PDF4 has a *RN* of 5.0 (Figure 2B). We also fitted the normalized filter capacity over the *RN* data using non-linear peak functions in Origin v9.1 (OriginLab, Northampton, MA). Maximum filter capacities were predicted for *RN* values of 5.40 (Gaussian), 5.49 (Lorentz), and 4.71 (Giddings). Adjusted R^2^-values of 0.47-0.49 indicated that all fits were in fair agreement with the data. A cubic fit to a previously published data set (adjusted R^2^ = 0.76) predicted an optimal *RN* of 3.37.

For filters with a normalized capacity ≥0.5, the concentrations of TSP (Figure 2C), DsRed and 2G12 (Figure 2D) fell within one standard deviation around the average observed for the PDF4 reference with F18 as the only exception, which contained less protein. Lower protein concentrations were observed in filtrates if the normalized capacity was below 0.5.

### Performance of small-scale devices with authentic filter geometry

**Figure 3:**
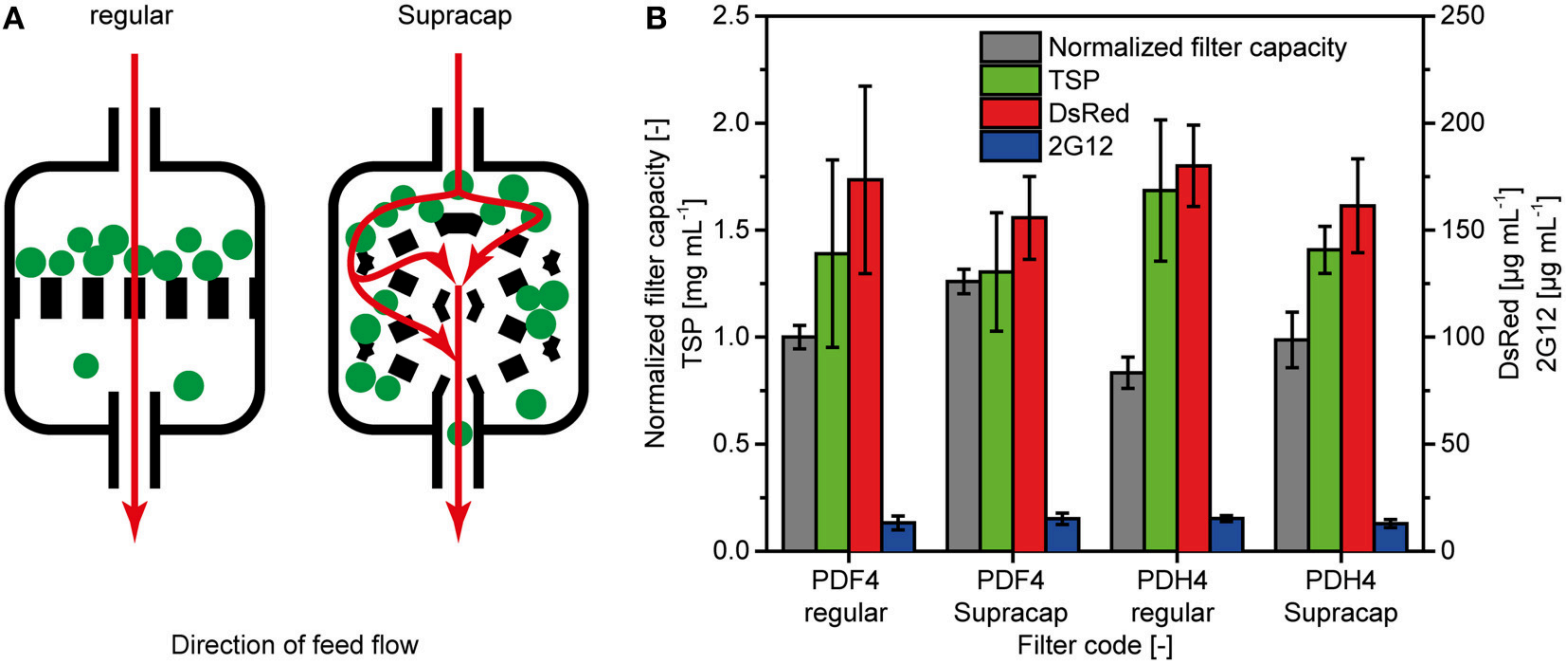
**Differences between depth filters with simple and authentic layer geometries**. **(A)** Schematic representation of the liquid flow pattern (red arrows) and particle deposition (green dots) on depth filters with simple geometry (regular) typically used during initial testing, and scaled-down devices with authentic layer conformation (Supracap). **(B)** Comparison of filter capacity and protein binding properties between regular and Supracap filter geometries. TSP, DsRed and 2G12 concentrations in filtrates were determined by the Bradford method, fluorescence spectroscopy and SPR spectroscopy, respectively. Error bars indicate standard deviations (*n* ≥ 3).

Small-scale filtration devices can have a layer geometry that differs from that used in process-scale equipment (Figure 3A). For depth filters PDF4 and PDH4, we have compared the effect of such different geometries on the filter capacity and protein binding efficiency using regular small-scale equipment (direct flow, regular) and devices mimicking the large-scale layer assembly (indirect flow, Supracap). The Supracap geometry increased the capacity of filter PDF4 significantly by 26% (two-sided *t*-test with 5% alpha level; Figure 3B), whereas an 18% increase was observed for filter PDH4, but this was not significant according to a two-sided *t*-test with a 5% significance level. There was no significant difference in TSP, DsRed or 2G12 concentrations among any of the four types of filter and geometry combinations (Figure 3B).

### Testing depth filters without diatomaceous earth

**Figure 4:**
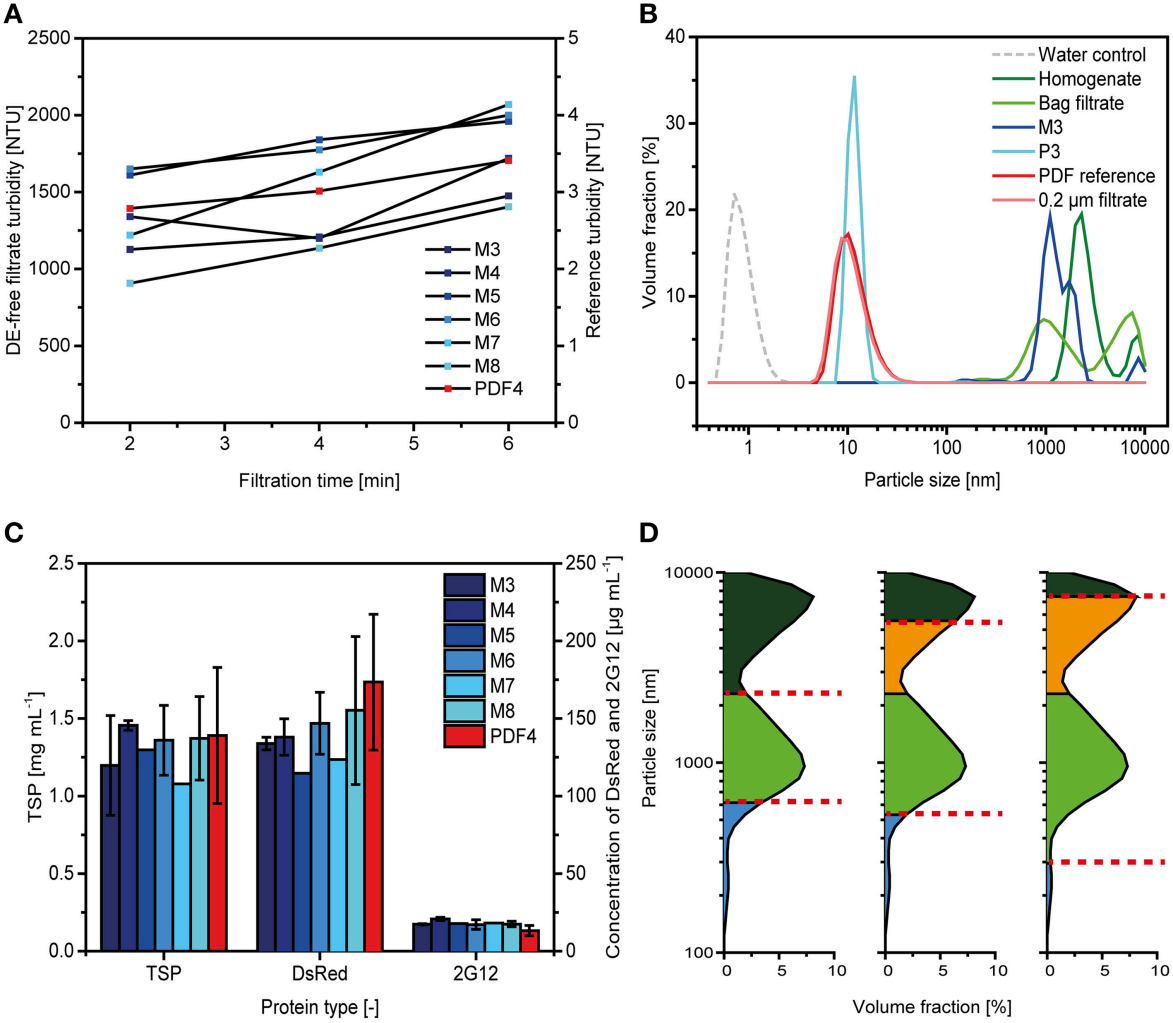
**Comparison of filters lacking diatomaceous earth with conventional depth filters**. **(A)** Depth filtrate turbidities observed during clarification for DE-free filters and a DE-containing reference. **(B)** Particle size distributions determined for selected depth filtrates compared to those observed for preceding (homogenate, bag filtrate) and subsequent (0.2 μm filtration) process steps. All measurements were taken with a Zetasizer NanoZS. **(C)** TSP, DsRed and 2G12 concentrations in different depth filtrates as determined by the Bradford method, fluorescence spectroscopy and SPR spectroscopy, respectively. Error bars indicate standard deviations (*n* ≥ 3). **(D)** Particle retention by depth filters selected based on actual particle size distributions (F6, left), prediction by *RN* (PDF4, middle) and empirical data (F14, right). Particles retained by the first and second filter layers are colored dark green and green, respectively. Particles in the filtrate are colored blue and those not retained on the first filter layer due to suboptimal selection of the retention rate are colored orange.

The DE-free filter P3 had the smallest retention rating of all the filters we tested here (Table 1) and also showed the lowest capacity of only 8 ± 1 L m^−2^ (*n* = 3). The other DE-free filters did not show any relevant increase in back pressure over the first 35 L m^−2^, i.e., the pressure level was ~0.02 MPa (0.2 bar; data not shown). Among these filters, M3 and M8 most effectively retained dispersed particles but filtrations were stopped after 35 L m^−2^ because in all cases turbidity was reduced only by a factor of 2-5 compared to the feed, a bag-filtered plant extract (Figure 4A). In contrast, P3 reduced the turbidity to 2 ± 1 NTU (*n* = 3), corresponding to a 1000-fold reduction and was similar to that of the reference filter PDF4.

The particle size distribution revealed that the P3 and PDF4 filtrates almost exclusively contained particles in the 10–20 nm range, similar to that observed for a 0.2 μm filtrate (Figure 4B). In contrast, the majority of particles in the M3 filtrate had a size of 1000-2000 nm, similar to the dominant particle populations in the bulk plant homogenate and bag filtrate.

We did not observe significant differences (two-sided *t*-test with 5% alpha level) in the TSP and DsRed concentrations of the different depth filtrates regardless of the presence or absence of DE (Figure 4C). The concentrations of antibody 2G12 were on average 38 ± 11% (*n* = 18) higher in filtrates from the DE-free filters compared to the DE-containing PDF4 reference. But this difference was only significant when comparing M4 and PDF4 (two-sided *t*-test with 5% alpha level). Furthermore, when we expressed the 2G12 concentration in the depth filtrate as a percentage recovery of the bag filtrate (the feed stream to the depth filter), we found that there was no difference between PDF4 and the DE-free filters given the recovery values of 85 ± 10% (*n* = 10) and 83 ± 9% (*n* = 18), respectively.

### Clarification of crude plant extracts by pre-coat filtration

**Figure 5:**
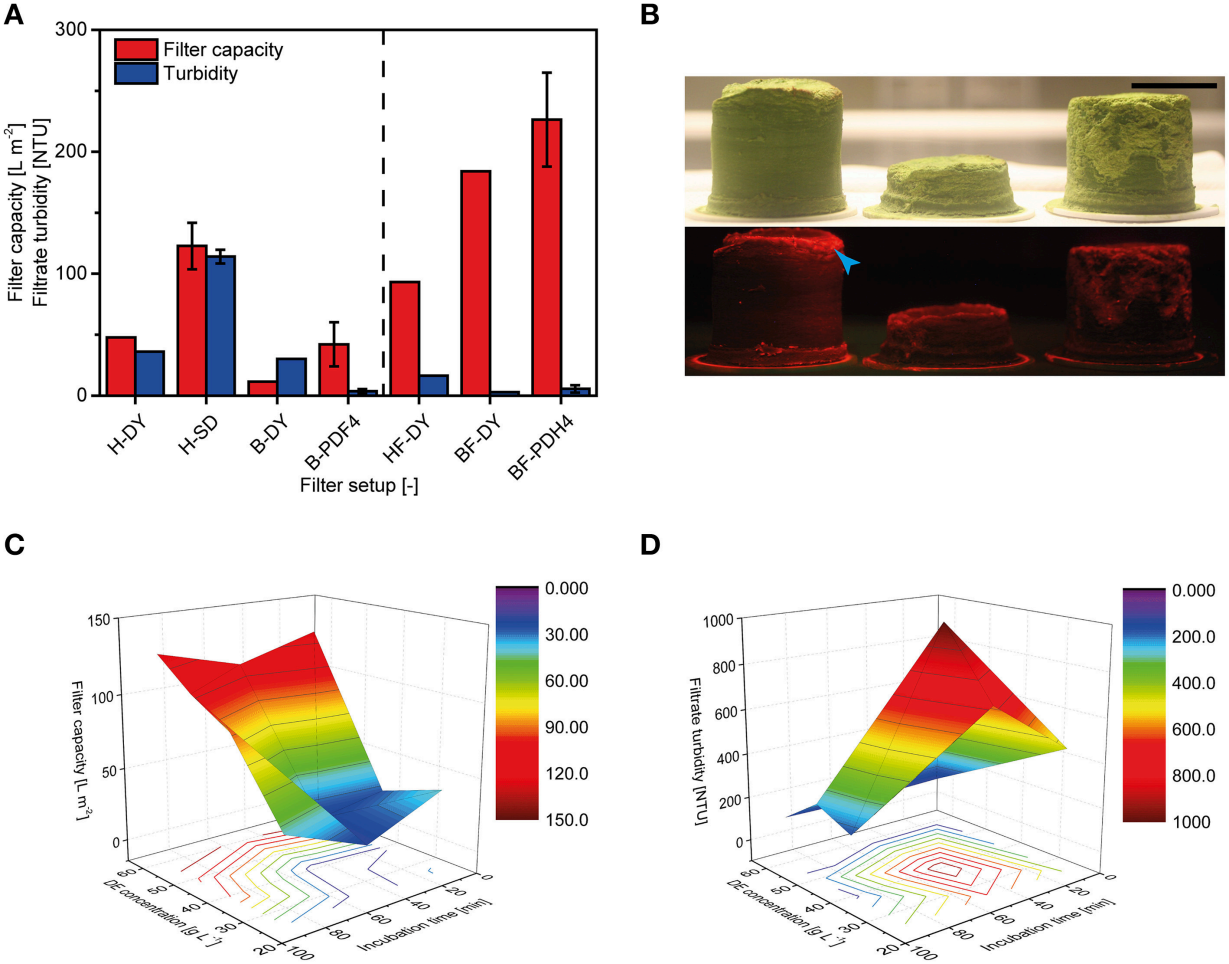
**Clarification of plant extracts using pre-coat filters**. **(A)** Filter capacity and filtrate turbidity achieved using bottle-top (DY) and small-scale filter devices (SD) fed with bulk plant homogenate (H) or bag filtrate (B). Results shown to the right of the dashed line originate from experiments in which 2.0 g L^−1^ Polymin P was added to the homogenate prior to further processing. Error bars indicate standard deviations (*n* ≥ 3). **(B)** Photographs of filter cakes under ambient light (top) or green light with red filter (bottom) obtained from SD filters using 50 (left), 25 (middle), or 40 (right) g L^−1^ DE. Red fluorescence originating from the target protein DsRed is highlighted with a blue arrowhead. Size bar = 30 mm. **(C)** The dependence of SD filter capacities on DE concentration and incubation time, from DoE runs. **(D)** The dependence of SD filtrate turbidities on DE concentration and incubation time, from DoE runs. The number of DE additions did not affect filter capacity or filtrate turbidity in the experiments shown in **(C,D)**.

Pre-coat filtration has the potential to replace bag and depth filter trains with a single unit operation (Figure 1C). Experiments were conducted with bottle-top devices for initial screening (DY) and then small-scale units with a head space geometry and filter pore size that matched those used in large-scale operations (SD). When bulk plant homogenate was loaded onto the DY filters (H-DY) a capacity of ~50 L m^−2^ was achieved, which matched the capacity of the PDF4 reference filter (B-PDF4) combined with the upstream bag filter. When DY filters were fed with plant extract that had already passed through the bag filter, the capacity dropped to ~10 L m^−2^. This feed-dependent difference in capacity was reversed if Polymin P was added to the homogenate, i.e., DY filters challenged with the flocculated homogenate (HF) exhibited a lower capacity than the same filters challenged with flocculated bag filtrate (BF) or a PDH4 depth filter control (Figure 5A). Using bag filtrate instead of homogenate, or including a flocculant, reduced the filtrate turbidity after DY, but none of these setups achieved the reduction in turbidity possible with conventional depth filters (4 NTU).

The use of pre-coat filtration directly after homogenization is most advantageous from a process design point of view as discussed below (Figure 1C). We therefore used a DoE approach to investigate the performance of pre-coat filters at this process step in more detail, using filter housings with authentic geometry and under varying process conditions. Increasing the concentration of DE in the feed stream increased the thickness of the resulting filter cake (Figure 5B) but did not accelerate filter blocking at any of the DE concentration we tested. DE concentrations of 50-60 g L^−1^ resulted in filter capacities of ~125 L m^−2^, which was a 2.5-fold higher than that of the PDF4 reference (Figures 5A,C). Reducing the DE concentration resulted in lower filter capacities. However, it was possible to compensate for the lower DE concentration by increasing the pre-filtration incubation times for DE and the homogenate to more than 60 min. Adding DE in steps to achieve the final DE concentration in the homogenate did not affect the filter capacity (data not shown).

The turbidity of the SD filtrates was reduced by 80% compared to the homogenate by adding 25-40 g L^−1^ DE. In contrast, we observed turbidities of 114 ± 6 NTU (*n* = 3) if we added 60 g L^−1^ DE to the homogenate, corresponding to a 50-fold reduction compared to the homogenate alone (Figure 5D). The turbidity declined during filtration from initial values of ~500 to 30 NTU at the end of the process.

The use of DE during pre-coat filtration did not affect protein concentrations and recoveries of 103 ± 17, 112 ± 26, and 97 ± 15% (*n* = 14 in all cases) were observed for TSP, DsRed, and antibody 2G12, respectively.

## Discussion

### Particle size distribution is more accurate than RN for the prediction of filter capacity

The capacity of 36 ± 6 L m^−2^ we observed for the PDF4 reference filter was in good agreement with the 37 ± 3 L m^−2^ reported in a previous publication that established this filter as a standard for plant extract clarification (Figure 1B; Buyel and Fischer, 2014c) confirming the comparability of the results presented here with preceding studies. Five of the depth filters we tested here showed similar or higher filter capacities compared to the PDF4 reference. The average *RN* of these filters was 5.9 ± 2.7 (*n* = 5) apparently confirming that selecting PDF4 with an *RN* of 5.0 as the standard depth filter was a reasonable choice to achieve high filter capacity given the data available from a series of filtration experiments. However, the highest capacity was achieved with a filter that had an *RN* of only 1.73 (F6) showing that predictions based on *RN* calculations can be inaccurate. This reflects the fact that predictions based on *RN* are only descriptive in nature, even though they are based on empirical results. In contrast, particle size analysis can reveal the actual distribution of dispersed species in a feed solution and allow the selection of filter layers based on mechanistic considerations. For example, solutions with a bimodal particle distribution can be clarified using two filter layers that have nominal retention ratings corresponding to the lower end of each mode. The use of filter layers with the coarsest applicable retention rate in each mode avoids premature pore blocking. We have observed such bimodal particle distributions in bag-filtered plant extracts with peaks at 0.95 and 7.50 μm. Figure 4D illustrates how filter F6 improved particle retention compared to the standard PDF4 filter and a suboptimal alternative, like F14, based on such a mechanistic description. This also explains why each of the best-performing depth filters had in common a second layer with a retention rating of ~0.6 μm, which effectively removed particles representing the peak at 0.95 μm. Particle size distributions can therefore be used to facilitate filter layer selection in a rapid and cost-effective manner, but *RN* can function as a substitute if particle size distribution data are unavailable.

Depth filter capacities of up to 1000 L m^−2^ have been reported recently (Buyel et al., 2014) but the performance in those experiments depended on two additives, a polyethylenimine-based flocculant and a cellulose-based filter aid. Although these improve filter efficiency, the flocculant can be incompatible with subsequent DSP steps (Buyel and Fischer, 2014b) and the cellulose-based filter aid can generate unacceptable amounts of dust (U.S. Department of Health and Human Services, 1995). It is therefore better to optimize depth filter capacities without additives if possible, simply by selecting filter layers with nominal retention ratings matching the particle size distribution in the feed stream. This also helps to reduce production costs and improve compatibility with subsequent DSP steps. There was no significant difference among the five filters with the highest capacities in terms of TSP, DsRed or 2G12 concentrations, confirming that filters from different vendors perform equally well in this respect and process optimization can focus on filter capacities. The reduced protein concentrations observed in the filtrate from other filters with low capacities can be attributed to a dilution effect resulting from residual rinse buffer that is retained in the filter layers after mandatory initial flushing. Based on our experience, this holdup buffer volume is 8-10 L m^−2^ for PDF4 and can thus reduce protein concentrations by 50% if the filter capacity is only ~10 L m^−2^, e.g., for filter F1. These data agree with the 53% reduction in TSP we observed in the filtrate produced by filter F1 compared to PDF4.

### Filter capsules with authentic geometry yield scalable filter capacity values

Filter capsules with authentic geometry channeled the feed stream onto the filter layers indirectly (Figure 3A) which probably delayed pore blocking and explained the ~20% capacity increase compared to the direct stream in conventional small-scale devices. We have used filter PDF4 in a GMP-compliant 800-L scale production process for monoclonal antibody 2G12 and found that the normalized filter capacity was 1.25 ± 0.17 (*n* = 3) compared to regular small-scale filters. This is in good agreement with the value of 1.26 ± 0.06 (*n* = 3) we calculated for the small-scale devices with authentic geometry (Supracap) reported above. Therefore, small-scale filtration devices with authentic geometry can achieve scalable depth filter capacities when filters are challenged with plant extracts during early process development. This will facilitate the estimation of production costs based on small-scale data and thus allow the economic evaluation of different process alternatives during the early development phase, ultimately improving the competitiveness of plant-based protein expression systems.

### Depth filters lacking diatomaceous earth do not reduce the turbidity of plant extracts

Among the DE-free filters we tested, only P3 reduced turbidity by the same amount as the PDF4 reference but this device also offered the lowest filter capacity. The turbidity in the other filtrates was >1000 NTU and thus not compatible with subsequent DSP steps including 0.2-μm filtration and chromatography. One major issue was that green pigments and particles of ~1 μm size passed through all of the DE-free filters except P3. The second layer of these filters had a nominal retention rating of 0.85 μm, which is 30-40% wider than the coarsest second layer of DE-containing filters yielding turbidities of 16 ± 7 (*n* = 5) NTU (0.65 μm second layer) or 4 ± 2 (*n* = 10) NTU (0.60 μm second layer). The average four-fold difference in the filtrate turbidity of DE-containing filters with second layers of 0.65 and 0.60 μm indicates that this size range marks a limit above which large numbers of particles begin to pass through the filter layers. These data support the particle size distributions we observed for the bag-filtered plant extract loaded onto DE-free and DE-containing filters, which had a first peak of dispersed particles with an average size of 0.95 μm (Figures 4B,D). It may still be possible to achieve low filtrate turbidity in combination with high filter capacity if new combinations of DE-free filter layers are used. For example, a combination of CE40 or CE30 as a first layer with Bio20 as a second layer would yield DE-free filters with nominal retention ratings of 2.0 + 0.7 μm and 3.75 + 0.7 μm, respectively, which is close the 2.25 + 0.65 μm combination of the best performing DE-containing filter, F6. However, the CE and Bio filter series are produced by different manufacturers and it is thus unlikely that a filter containing both types of layers will become commercially available for pharmaceutical-grade applications in the near future. We therefore did not test this combination.

There was no difference in the TSP and DsRed concentrations of filtrates that passed either DE-free or DE-containing filters. There was also no difference in the recovery of antibody 2G12 if concentrations were normalized to the corresponding plant batch, indicating that DE-free filters offer no improvement in yield for most tobacco HCPs and the two target proteins we tested. However, other target proteins may unexpectedly bind to DE-containing filters (our unpublished data) and the development of an effective clarification strategy using DE-free filters can thus be a worthwhile investment for future processes.

### Pre-coat filtration with diatomaceous earth simplifies the clarification process

In the absence of flocculants, DY pre-coat filters achieved higher capacities when fed with bulk plant homogenate instead of bag-filtered extract, even though the latter has a turbidity of 3000-6000 NTU, (Buyel and Fischer, 2014e) which is 30-80% less than that of the bulk homogenate (Buyel and Fischer, 2014f). This observation appears counterintuitive, but is probably explained by the presence of cellulose fibers and coarse cell debris in the bulk homogenate with a size range of 1000-4000 μm,(Buyel and Fischer, 2014b) which helps to form a filter cake and thus increases the filter capacity (Buyel et al., 2014).

The presence of flocculants increased the DY filter capacity when bulk homogenate was used as feed, but the effect was more impressive when bag filtrate was used instead. The 10-μm aggregates that are typically found in flocculated bag filtrate (Buyel and Fischer, 2014b) may help to form a more effective filter cake when combined with the Celpure C300 diatomite compared to the 1-8 μm particles in the untreated bag filtrate or the 1000-4000 μm particles found in the (flocculated) homogenate.

These results indicated that pre-coat filtration is most effective when applied directly after homogenization in the absence of flocculants because under these conditions the filter capacity was comparable to a reference depth filter but required only a single filtration step instead of bag and depth filters in series (Figure 1). This can simplify the clarification procedure and thus improve process control. It can also reduce the time and labor required for process setup and the cost of consumables. Furthermore, if a transient expression system is used, a single-use pre-coat filtration step would eliminate the need for reusable bag filter housing, eliminating the need for cleaning validation and the risk of product carryover (Figure 1B).

To confirm these anticipated advantages, we used SD filters that have the same geometry as large-scale production modules. When bulk plant homogenate was used as the feed, SD filtration increased the filter capacity by 2.5-fold compared to DY filters because the SD setup allowed a defined volumetric feed flow rate resulting in a gradual and thus more effective buildup of filter cake. In contrast, DY filtration requires the single-step application of DE-homogenate slurry to the filter and the flow rate is defined by the combined effect of the applied vacuum and the degree of filter blocking. Therefore, DY filters are useful for initial screening purposes, i.e., to investigate the general compatibility of pre-coat filtration with a given clarification requirement, but SD filters can be used to optimize filtration conditions and yield potentially scalable capacities due to their authentic geometry, e.g., they feature the same base filter porosity and head space as production modules.

Even so, the turbidity of the SD filtrates was higher than that observed for DY filters. This may reflect the lower base filter porosity of DY (0.2 μm) compared to SD (7-12 μm). Based on these retention ratings and the particle size distribution of the bulk homogenate (Figures 4B,D) a high turbidity in SD filtrates is expected. However, the turbidity of SD filtrate was still 10-fold lower than that of DE-free depth filters, which had retention ratings of 0.85 μm. This shows that the filter cake formed during SD filtration can effectively retain particles even in the 0.5-1.0 μm range. We found that turbidity declined during the course of SD filtration, suggesting that particle retention improved as the thickness of the filter cake increased, as previously reported (Cain, 1984; Smith, 1998). We have found that turbidities of up to 50 NTU are compatible with 0.2 μm filtration, achieving capacities exceeding 350 L m^−2^ (our unpublished data), but a turbidity of ~100 NTU as observed for SD filtration may interfere with subsequent DSP steps. One potential way to reduce the turbidity of the SD filtrate is to cycle the feed until a sufficient thickness of cake has accumulated. An initial cake build up phase during clarification has been included in other processes for this purpose (Smith, 1998; Cain, 1984). The protein concentrations of TSP, DsRed and antibody 2G12 were not affected by pre-coat filtration, indicating its general applicability during the manufacture of plant-derived biopharmaceutical proteins.

## Conclusions

In this study we show that analyzing the particle size distribution of plant extracts allowed the rational selection of depth filter layers achieving higher capacities than layers selected based on the previously suggested descriptive *RN* model. We also demonstrate for the first time that small-scale filters with authentic geometry provide reliable capacity data for filtration scale-up of plant-based processes using an 800-L GMP-compliant production scale process as a reference. Furthermore, we highlight that new DE-free depth filters hold the potential to improve product recoveries during the clarification of plant extract due to reduced protein binding and provide recommendations for the selection of the according filter layers. Finally, we underline that implementing a new pre-coat filtration strategy in the clarification procedure simplifies the process stream, reduces the number of unit operations and increases the compatibility of the primary processing with single use-technologies. These findings will help to increase the economic competitiveness of plant-based processes compared to traditional fermentation and cell culture approaches.

## Funding

This work was funded in part by the European Research Council Advanced Grant “Future-Pharma,” proposal number 269110, the Fraunhofer-Zukunftsstiftung (Fraunhofer Future Foundation) and the Frauhofer-Gesellschaft Internal Programs under Grant No. Attract 125-600164.

### Conflict of interest statement

The authors declare that the research was conducted in the absence of any commercial or financial relationships that could be construed as a potential conflict of interest.

## Abbreviations

DE: diatomaceous earth
DoE: design of experiments
DSP: downstream processing
DY: Sartolab DY
HCP: host cell protein
NTU: nephelometric turbidity units
RN: retention number
SD: Sartoclear dynamics
SPR: surface plasmon resonance
TSP: total soluble protein
USP: upstream production.
